# Evaluation of structured training in osteotomy and splitting for BSSO using a 3D-printed mandibular model

**DOI:** 10.1186/s12909-026-09748-w

**Published:** 2026-07-09

**Authors:** Leonhard Gerich, Yao Li, Oliver-Costin Vladu, Moritz Bregenzer, Kunpeng Xie, Jan Egger, Rainer Röhrig, Frank Hölzle, Behrus Hinrichs-Puladi, Ashkan Rashad

**Affiliations:** 1 Department of Oral and Maxillofacial Surgery, University Hospital RWTH Aachen, Pauwelsstraße 30, Aachen, 52074 Germany; 2 Institute of Medical Informatics, University Hospital RWTH Aachen, Aachen, 52074 Germany; 3 Institute for Artificial Intelligence in Medicine, University of Duisburg-Essen, Essen, 45131 Germany; 4 Department of Oral and Maxillofacial Surgery, GFO Kliniken Mettmann-Süd, St. Josefs Krankenhaus Hilden, Hilden, 40724 Germany

**Keywords:** 3D-printing, Medical education, Orthognathic surgery, Bilateral sagittal split osteotomy

## Abstract

**Background:**

The teaching of bilateral sagittal split osteotomy (BSSO) requires the utilisation of practical training methods to optimise the surgical result and prevent complications such as bad splits or damage to the inferior alveolar nerve. The objective of the present study was to evaluate a structured training program using a cost-effective, 3D-printed mandibular model to improve osteotomy and splitting performance in BSSO.

**Material & methods:**

The study was registered in the German Clinical Trials Register (DRKS00034369, registration date: 20/11/2024). A lower jaw model was fabricated using a semi-professional Prusa XL Multi-Tool 3D printer and a combination of colours and materials. The model also incorporated the inferior alveolar nerve, which was fabricated from a flexible material. The model was modified to facilitate its dynamic integration into a phantom head by modification of the condyles. Twenty participants performed two repetitions of the osteotomy and splitting steps of BSSO. The participants received feedback after the first run and during the second run. The experiments were recorded and assessed using a validated questionnaire (OSATS). Furthermore, the distance between the inferior alveolar nerve and the fracture surface, as well as the number of bad splits, was determined.

**Results:**

Participants significantly improved their surgical skills as measured by the OSATS between the first and second run from 23.7 points ± 4.3 (mean ± sd) to 27.2 points ± 3.9 (*p* < 0.001). In addition, the minimal distance between the nerve and the fracture surface increased from 0.2 ± 0.38 mm to 1.12 ± 1.04 mm (*p* = 0.012). The number of bad splits decreased from 15 to 4 (*p* = 0.044). The estimated material cost of the lower jaw is approximately US$4 with a printing time of an estimated 9.5 h.

**Conclusion:**

It is possible to develop a cost-effective and realistic training model, that can be used within structured repeated practice sessions for BSSO training and is associated with improved performance in this setting. Further studies are needed to validate the simulator and assess its impact on real-life surgical outcomes.

**Supplementary Information:**

The online version contains supplementary material available at 10.1186/s12909-026-09748-w.

## Introduction

Repositioning osteotomies, particularly bilateral sagittal split osteotomies (BSSO), are a well-established surgical approach in the interdisciplinary treatment of congenital and acquired jaw malocclusions [17]. Due to the anatomical complexity of the mandibular region and the need to fulfil functional and aesthetic criteria, these procedures require a high level of surgical expertise [25]. Despite their routine use, complications remain a significant concern, with bad splits and injuries to the inferior alveolar nerve (IAN) being the most notable [29, 32, 46]. Damage to the IAN can result in neurosensory deficits that severely impact patients’ quality of life [7].

To minimise such risks, a structured learning process involving repeated training is essential, particularly when acquiring BSSO-specific competencies [2, 8, 43]. Cadaver-based training is often considered the gold standard due to its unmatched anatomical and biomechanical realism [5, 51]. However, cadavers are costly, logistically challenging and often limited in availability [14, 35]. Consequently, there is an increasing need for alternative training tools that allow reproducibility and realism while reducing cost and logistical effort. Commercially available models, such as those from Synbone priced at approximately 21–35 US $ per model, offer limited realism: Depending on the design, they differentiate between compact and cancellous bone, but lack integration of the IAN into the mandible [9]. Furthermore, these models are made of a single material, are single-coloured, offer only limited integration into a phantom head and cannot be adapted individually [9].

3D-printed models, such as those described by Bertin et al. and Yoshida et al. have successfully enhanced both the theoretical understanding and the surgical technique [6, 52]. However, these studies relied on the costly PolyJet printing process, costing around US155 per mandible and excluded the IAN or intraoperative feedback. Furthermore, they lacked structured evaluation systems and opportunities for repeated training under expert supervision and with expert feedback, which are key factors in long-term skill development [11, 31, 34, 47]. 

Recent studies, such as Seifert et al. have presented one of the most advanced 3D-printed training models currently described in the literature for orthognathic surgery, including features such as a mucosal mask to enhance realism. However, these authors also report limitations, including the lack of objective performance assessment and the absence of a representation of the IAN. Reported model costs of approximately US$200 (€180) per unit reflect the level of anatomical and technical sophistication of such systems [39].

Emerging technological advances now enable the production of multi-material 3D-printed models using cost-effective Fused Deposition Modelling (FDM) printers, such as the Prusa XL (Prusa Research, Prague, Czech Republic). These semi-professional printers can process up to five different materials simultaneously, enabling visual and tactile differentiation between cortical bone, cancellous bone, teeth and the IAN. Consequently, realistic and functional jaw models can now be produced at a fraction of the previous cost, while maintaining a high degree of anatomical accuracy and visual realism [53].

This study aimed to develop and assess a cost-effective, multi-material 3D-printed lower jaw model for realistic simulation of osteotomy and splitting in BSSO procedures. Key features included high anatomical fidelity and surgical functionality, as well as a flexible and visually distinct IAN. A structured training protocol involving repeated supervised osteotomies, intraoperative feedback and postoperative evaluation via questionnaires and 3D-scanning was applied to examine whether the training environment improves surgical skill and anatomical awareness in BSSO training.

## Materials and methods

### Study design

A total of 20 participants were included in the study, comprising 10 dental students in clinical semesters from RWTH Aachen University (Germany) and 10 dentists and medical doctors from the Department of Oral and Maxillofacial Surgery of the University Hospital RWTH Aachen. The primary endpoint of the study was improvement in the participants’ surgical skills, as measured by the validated OSATS assessment tool [10]. The secondary endpoints included evaluating the participants’ understanding of the BSSO procedure, determining the distance from the fracture gap to the IAN and assessing the visual and haptic perception of the model, as well as its practical suitability. Additionally, the incidence of bad splits was quantified and discrepancies between the responses of students and doctors were identified. The study was reviewed and approved by the RWTH Aachen University ethics committee (approval number EK 24–319, approval date 10/09/2024 by Prof. Dr. G. Schmalzing), and the study protocol was registered with the German Clinical Trials Register (DRKS00034369, registration date 20/11/2024). The trial was successfully conducted between 7 December 2024 and 19 January 2025 in the Department of Oral and Maxillofacial Surgery at University Hospital RWTH Aachen, Germany.

### Sample size calculation

A sample size calculation was performed using G*Power (version 3.1.9.7). The calculation was based on a minimal expected effect size of 5 points on the GRS between pre- and post-training assessments using a 3D-printed training model. Assuming a standard deviation of 5.36 [10], a significance level of α = 0.05, and a power of 95%, the required sample size was calculated for a Wilcoxon signed-rank test. This resulted in a required sample size of 16 participants. To account for potential dropouts and non-evaluable datasets, an additional four participants were included.

### Model development

**Fig. 1:**
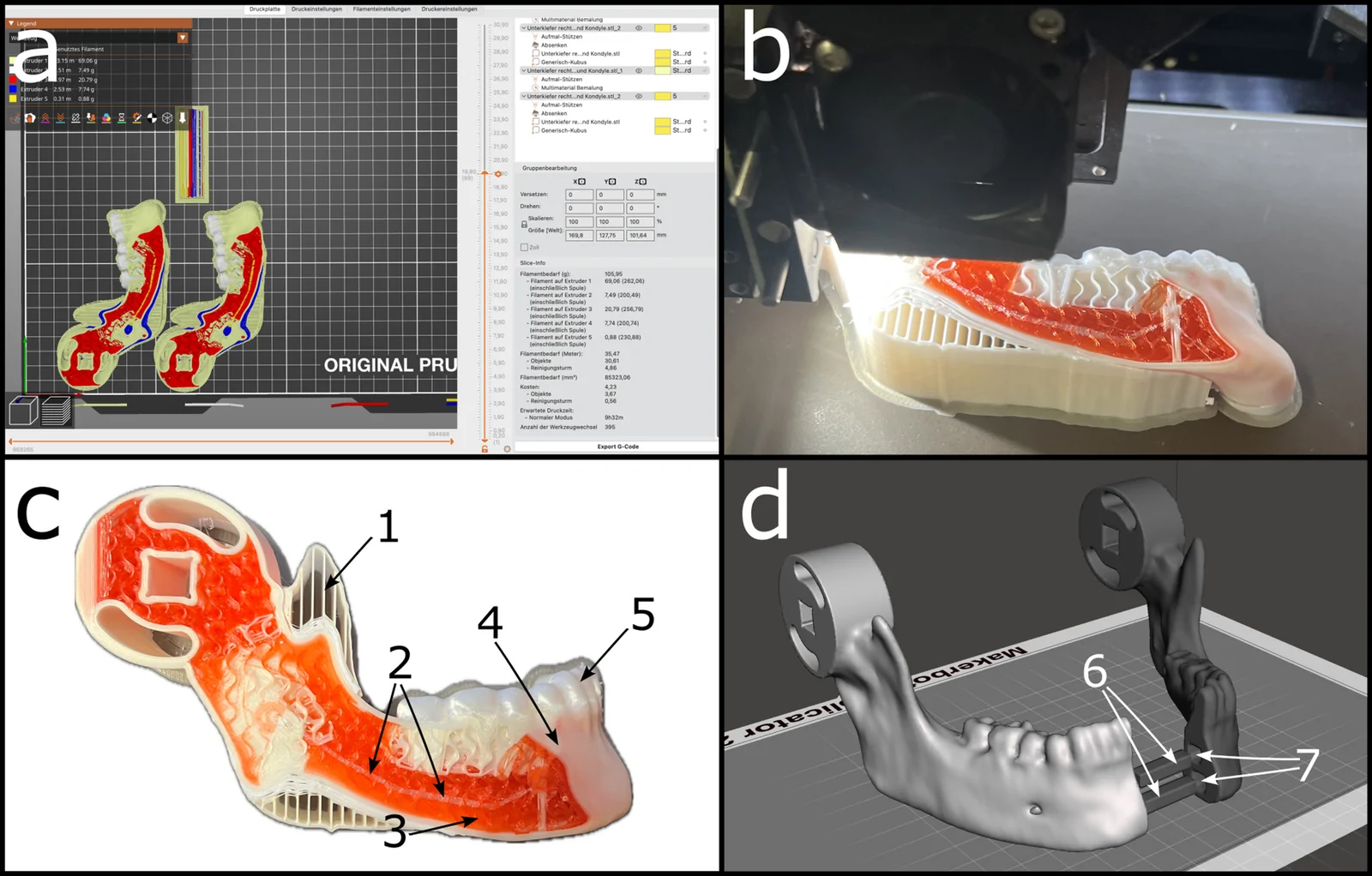
**(a)** Planned model in PrusaSlicer to determine printing orientation on the printing bed and the different materials for the corresponding structures. **(b)** Model during printing process. **(c)** A mandibular model that has been partially printed to show its different layers and inner structures: (1) support material; (2) inferior alveolar nerve; (3) cancellous bone; (4) cortical bone; (5) teeth. **(d)** Plug connection (6) designed in Meshmixer that can be inserted into the corresponding openings of the two jaws (7) so that the left and right halves are firmly connected to each other

The model was derived from a publicly available database of high-resolution CT scans of healthy skulls [22]. To recreate the IAN in a different material, both the nerve and the mandible were processed using the 3D Slicer [13]. The mandible (model: A0028) was originally provided as a pre-segmented model from the database and is available under a CC BY 4.0 license, which was imported into 3D Slicer and subsequently refined through manual corrections where necessary (segmentation: L.G.; quality control: B.HP.). The IAN was not directly segmented from the CT data but instead manually reconstructed slice-by-slice based on the course of the mandibular canal to ensure anatomical accuracy. The STL-file was then imported into the 3D animation suite Blender (version 3.6 LTS, www.blender.org) to modify the condyles for later implementation in a phantom head. Assembling a model composed of two jaw halves required conceiving a plug connection in Autodesk Meshmixer (version 3.5, www.meshmixer.com) to firmly fix the halves together (Fig. 1).

To achieve the most realistic and vivid representation possible, the three outer layers simulating cortical bone were printed in bone-coloured PLA (Prusament PLA Vanilla White) (Fig. 1). The gyroid-shaped 15% infill was fabricated from transparent red PLA/PHA (ColorFabb PLA/PHA) to accentuate the bone marrow structure. The teeth were printed in white PLA (Prusament PLA Blend Pearl White) and the IAN was made from flexible TPU (Flexfill TPU 92 A Natural, 1.75 mm) to ensure a realistic level of elasticity. Due to limitations of the slicing workflow implemented in PrusaSlicer, it was not possible to reproduce a distinct cortical canal layer in the present model. Instead, the IAN was embedded within the cancellous structure. PETG (Prusament PETG Signal White) was chosen as the support material as it can easily be detached from PLA. The plug-in device connectors were printed in PC (Polymaker PolyMaxTough, 1.75 mm). The printing process was facilitated by the Prusa XL Multi-Tool. The model was printed in layers, each with a thickness of 0.2 mm. This generated predetermined breaking points along the length of the filament within the model. To induce a controlled fracture during the exercise, it was crucial to align the mandible on the print bed so that the lower and posterior edges of the mandibular ramus ran parallel to it. The mechanical properties of the model were assessed through expert validation by an experienced oral and maxillofacial surgeon (O.V.), with a high annual volume of BSSO procedures. The validation was based on hands-on use of the printed model with standard surgical instruments in a simulated BSSO scenario. The surgeon evaluated the model qualitatively in terms of haptic feedback, resistance during cutting and splitting, and overall realism compared to clinical experience. Feedback was collected based on predefined aspects of tactile impression and mechanical behaviour. This expert assessment was used to confirm the suitability of the material properties for the intended training purpose. A prefabricated phantom head (P-5/3, Frasaco, Germany) was customised in the scientific workshop at University Hospital RWTH Aachen. The phantom head enabled both the upper and lower jaw to be clamped. Rotation of the lower jaw around the temporomandibular joint axis can be achieved by loosening and tightening two screws, allowing for a maximum angular displacement of 60°.

### Training session and evaluation

**Fig. 2:**
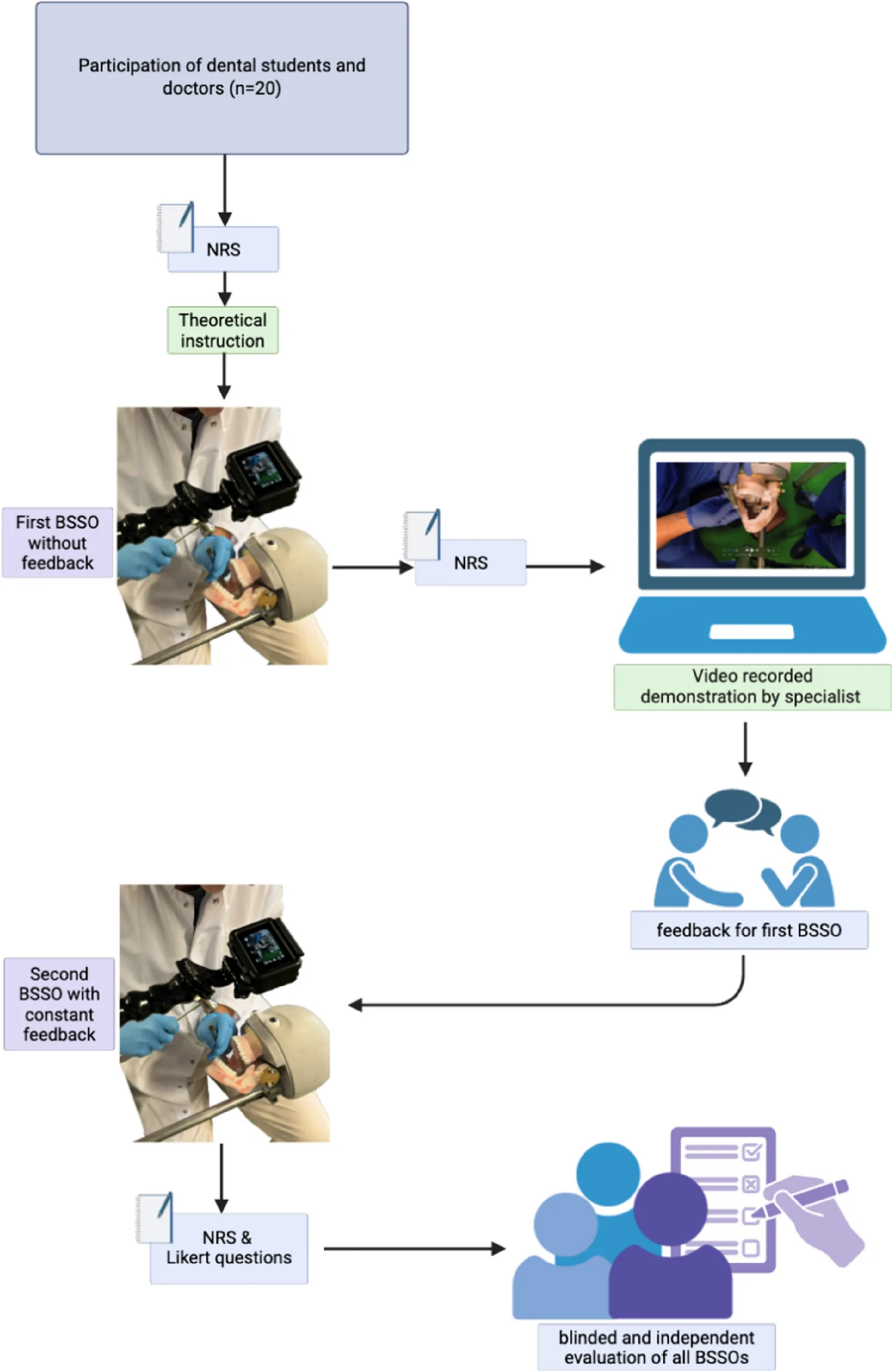
Description of the study course. NRS = Numeric Rating Scale

**Fig. 3:**
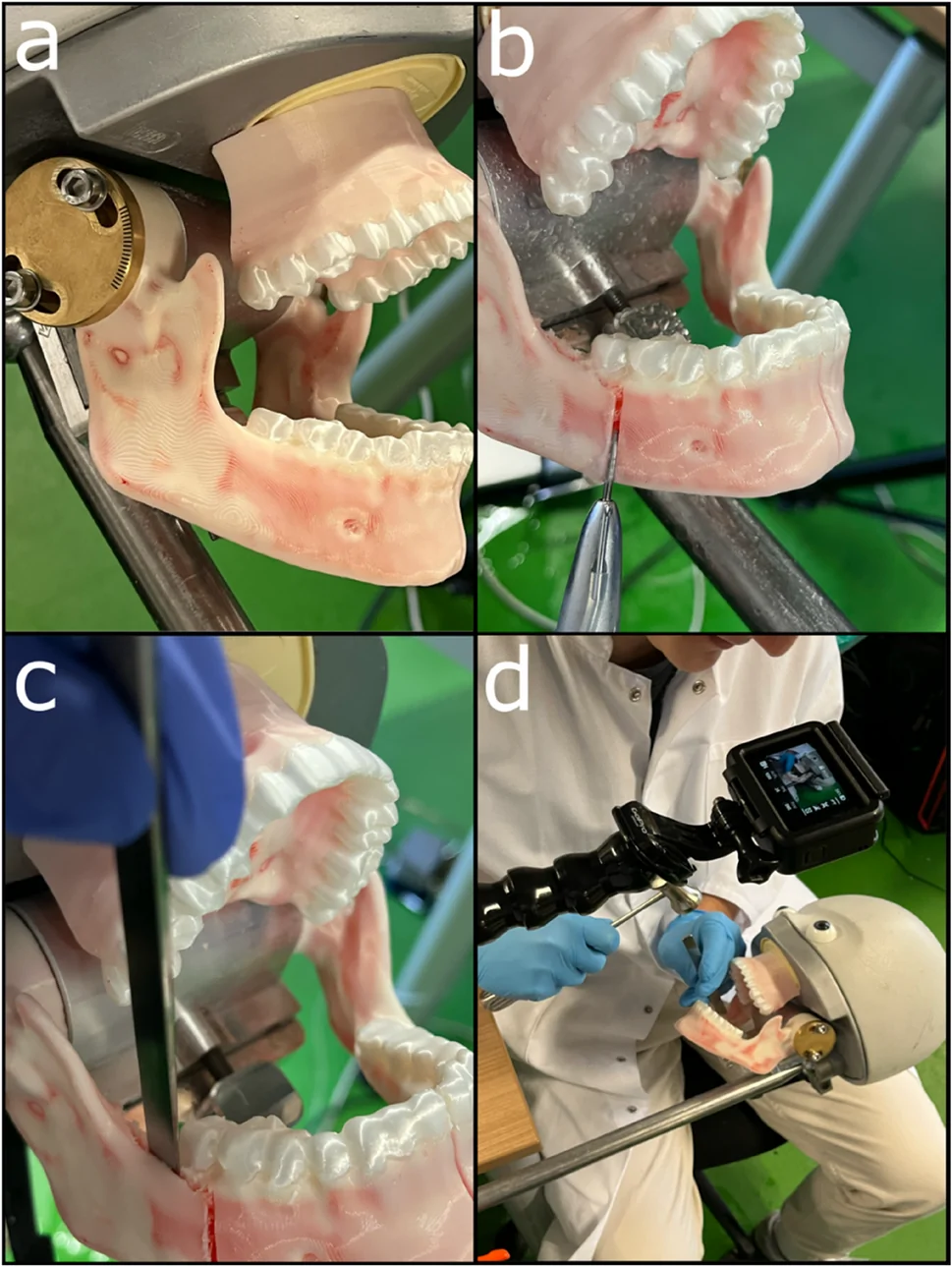
**a **The clamped lower jaw model can be rotated around the condyles in the phantom head and fixed in position using two screws. The head itself can also be moved freely. **b** A participant performing the osteotomy of the mandible with a Lindemann bur. **c** A participant performing the splitting of the mandible along the cortical osteotomy line with a hammer and chisel. **d** Experimental setup with participant performing the mandible splitting and video recording of the working area

The study participants were entrusted with implementing and evaluating the BSSO training (Fig. 2). Following the collection of demographic data, participants’ subjective experience in surgical skills and anatomical knowledge was assessed at the beginning of the training programme using a 10-point Numeric Rating Scale (NRS; 1 = very low experience, 10 = very high experience). Ratings were obtained across five domains: required surgical equipment, identification of anatomical landmarks, use of a Lindemann bur, sagittal splitting of the mandible and management of the IAN. This was followed by theoretical instructions in text and image form on how to perform the Dal Pont surgical technique [33]. The participants then began the exercise on the right side. After executing the osteotomy using a surgical handpiece (KaVo Intrasurg 1000, KaVo Dental GmbH, Biberach/Riß, Germany) and a Lindemann bur (H254E, Komet/Gebr. Brasseler GmbH & Co. KG, Lemgo, Germany), the tooth-bearing part was then separated from the joint-bearing part using a hammer and chisel (Fig. 3).

Osteosynthesis of the parts in new occlusion was neither performed nor part of the study. The participants were asked to complete the NRS once again. They then observed an experienced orthognathic surgeon (O.V.) performing the BSSO on a phantom head via a recorded video with audio. The BSSO procedure was evaluated using the processed model and suggestions for improvement were made by the study supervisor (L.G.). The participants then repeated the osteotomy and splitting on a new model on the same side, this time receiving constant feedback on the surgical technique. Finally, they answered the NRS questions again and assessed the functional and visual characteristics of the mandibular model using a Likert questionnaire. The Likert-scale questionnaire consisted of four response options (“strongly disagree”, “rather disagree”, “rather agree”, and “strongly agree”), coded on a 4-point Likert scale (1 = strongly disagree to 4 = strongly agree).

To subsequently evaluate surgical skills using a validated questionnaire, the participants’ performance during the exercise was recorded in their working area (GoPro Hero 5, GoPro Inc., San Mateo, California, USA) (Fig. 3). The blinded and independent evaluation, based on the video material, was carried out by two consultants (O.V. and A.R.) and a specialist (B.HP.). The Global Rating Scale (GRS) of the OSATS was used as the evaluation tool [10]. This tool enables objective evaluation of participants’ skills in the following domains, irrespective of the surgical procedure performed: respect for tissue; time and motion; instrument handling; knowledge of instruments; flow of operation; use of assistants; knowledge of specific procedure; and overall performance. The total score for each of the eight GRS evaluation criteria was calculated, and the mean value for the three evaluators was determined.

### Model analysis via 3D scan

**Fig. 4:**
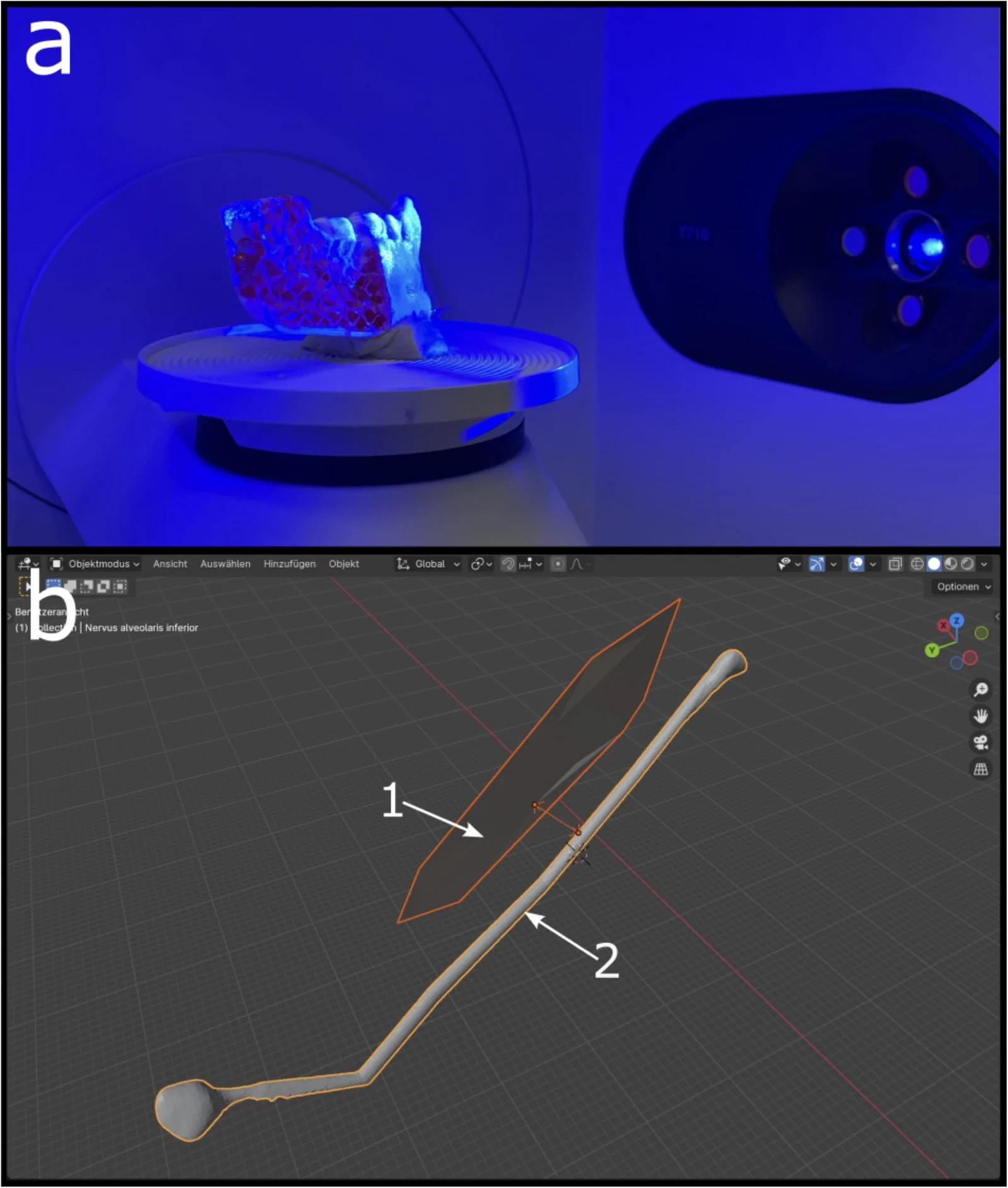
**a** Split mandible model during 3D-scanning fixed with modelling clay using a 3D-Scanner (Medit T710, Medit, Seoul, South Korea). **b** Distance measurement in Blender between the designed split surface (1) and the inferior alveolar nerve (2) via a python script

To ascertain the minimum distance between the fracture surface and the IAN, the tooth-bearing parts of the split models were scanned using the T710 3D scanner (Medit, Seoul, South Korea) at a resolution of 4 μm (Fig. 4). As the scanner operates using phase-shifting optical triangulation, light reflections and artefacts must be avoided. Therefore, a thin layer of matt occlusion spray (Okklusol Spray white superfine, Dentalligent GmbH, Remagen, Germany) was applied to the shiny fracture surface. The resulting layer thickness, measured in the range of a few micrometers, can be considered negligible in relation to the precision of the distance measurement. To align the scanned split models with the planned unsplit models, a two-phase, two-stage rigid registration pipeline was implemented using Open3D [54]. In the first phase, both the scanned and planned meshes were converted to point clouds and down-sampled using a voxel size of 5 mm. Fast Point Feature Histograms (FPFH) were computed to perform coarse alignment via RANSAC-based global registration as the first stage, followed by point-to-plane Iterative Closest Point (ICP) refinement as the second stage. This initial registration served to align the scanned models approximately to the same spatial position and orientation as the planned models. In the second phase, both meshes were segmented to retain only the anterior (unsplit) portion, reducing mesh discrepancies. The same registration procedure was then repeated using the initially aligned models as input. The final alignment achieved an inlier root mean square error below 0.4 mm and a fitness value exceeding 0.7. 

Using the Blender software, the original model was separated into nerve and jawbone components. The jawbone component was faded out so that the split jaw could be displayed with the correct nerve course. The fracture surface along the nerve was defined manually by two independent raters (L.G. and M.B.) by selecting all the constituent points, which were then connected to form a polygonal surface. Using a Python script, the shortest distance between the surface and the IAN was calculated (Fig. 4). The mean value for both evaluators was determined.

### Statistical analysis

A statistical analysis was performed using R (version 4.5.2, www.r-project.org). Due to the small sample size, non-parametric tests were employed. The GRS (the primary endpoint) was analysed using the Wilcoxon signed-rank test to report the mean difference with 95% confidence intervals (CIs). Students and doctors were compared using the Wilcoxon rank-sum (Mann-Whitney *U*) test. NRS measurements were analysed using the Friedman test and post hoc Wilcoxon signed-rank tests; the IAN distance was analysed using the Wilcoxon signed-rank test; and bad splits were analysed using McNemar’s test. Inter-rater reliability was assessed using the ICC (absolute agreement and consistency). Likert and open questions were analysed descriptively. The primary endpoint was tested as a single confirmatory hypothesis without correction, while all secondary and subgroup analyses were considered exploratory and adjusted using Holm’s correction. A post hoc subgroup analysis of doctors by experience (less than five years versus five years or more) was added during the review process and was left uncorrected given the small sample size.

## Results

A total of 20 participants were included in the study: 10 dental students in clinical semesters, 7 residents, and 3 specialists from the local Department of Oral and Maxillofacial Surgery. The students had a mean age of 22.3 years (sd ± 0.9) and were, on average, in their 9th semester of study (± 1.7). The doctors had an average age of 31.0 years (± 4.0) and an average of 4.9 years (± 3.1) of professional experience. On average, the doctors had previously performed 3.8 (± 6.5) bilateral sagittal split osteotomies (BSSOs).

### Global rating scale and self-assessment

**Fig. 5:**
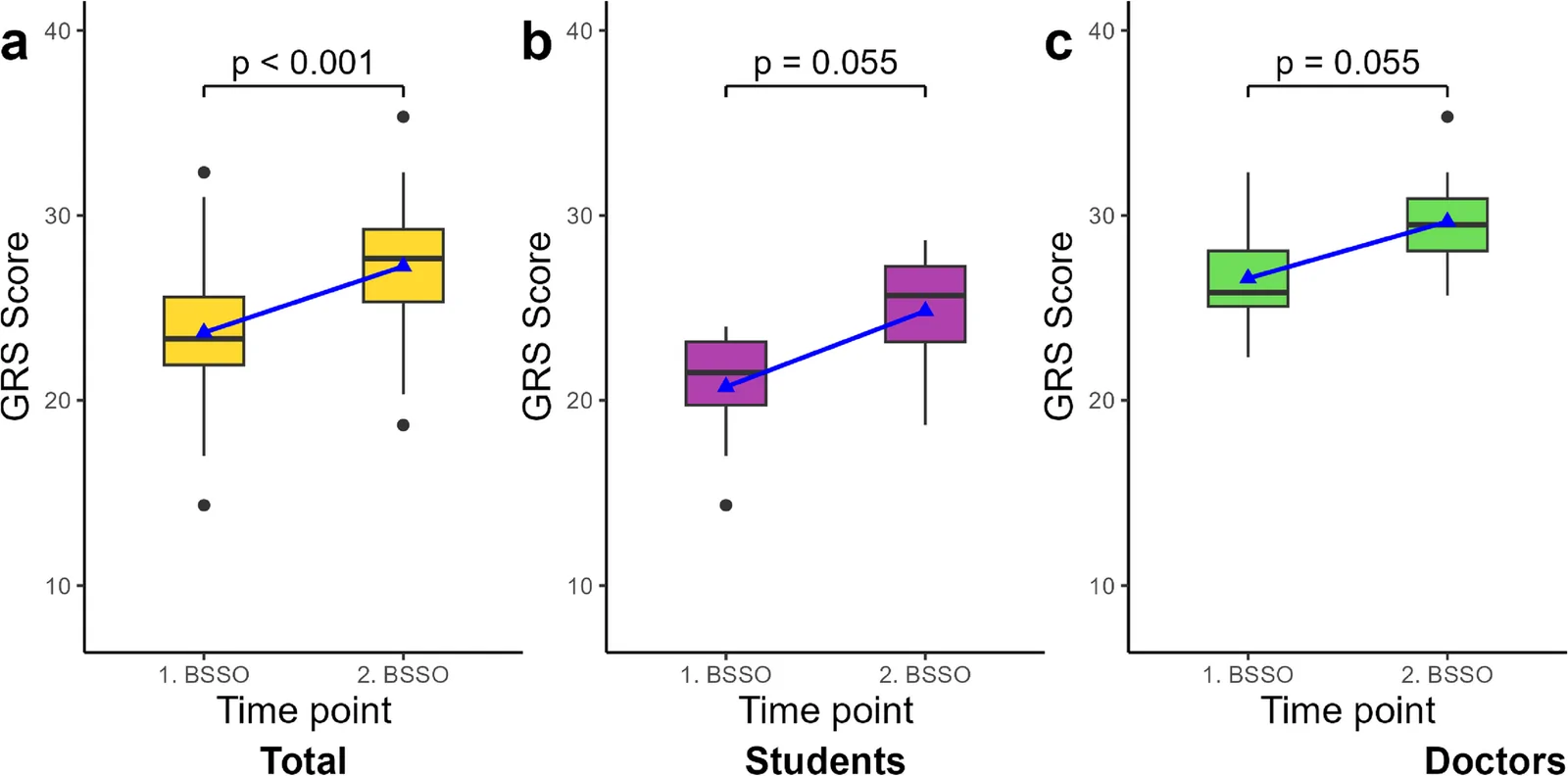
Results from the GRS of the two trials by **a** total (primary endpoint), **b** doctors and **c** students during the 1. BSSO and 2. BSSO. The results are based on the average GRS evaluations from the three independent investigators. Each boxplot shows the mean value (blue triangle). The black points mark the outliers. GRS = Global Rating Scale. The p-value (Wilcoxon signed-rank test) is shown; unadjusted for the primary endpoint and Holm-corrected for the subgroups

Evaluation of the GRS revealed significant improvements in participants’ performance (Fig. 5). On a scale ranging from 8 to 40 points, the mean difference (effect size) was 3.59 (95% CI 2.12–5.06), with participants achieving an average score of 23.7 (± 4.3) in the initial trial and 27.2 (± 3.9) in the subsequent trial (Wilcoxon signed-rank test, *p* < 0.001; Fig. 5a). Students demonstrated a mean improvement of 4.1 points, increasing from 20.7 (± 3.1) to 24.8 (± 3.4) (Wilcoxon signed-rank test, adj. *p* = 0.055; 95% CI 1.22–6.98; Fig. 5b). Doctors showed a mean improvement of 3.1 points, rising from 26.6 (± 3.2) to 29.7 (± 2.9) (Wilcoxon signed-rank test, adj. *p* = 0.055; 95% CI 1.63–4.53; Fig. 5c). Doctors achieved higher scores than students in both the first (Mann-Whitney *U* test, adj. *p* = 0.015) and second runs (Mann-Whitney *U* test, adj. *p* = 0.045). The intraclass correlation coefficient (ICC) for absolute agreement was 0.60, whereas the ICC for consistency was 0.75.

**Fig. 6:**
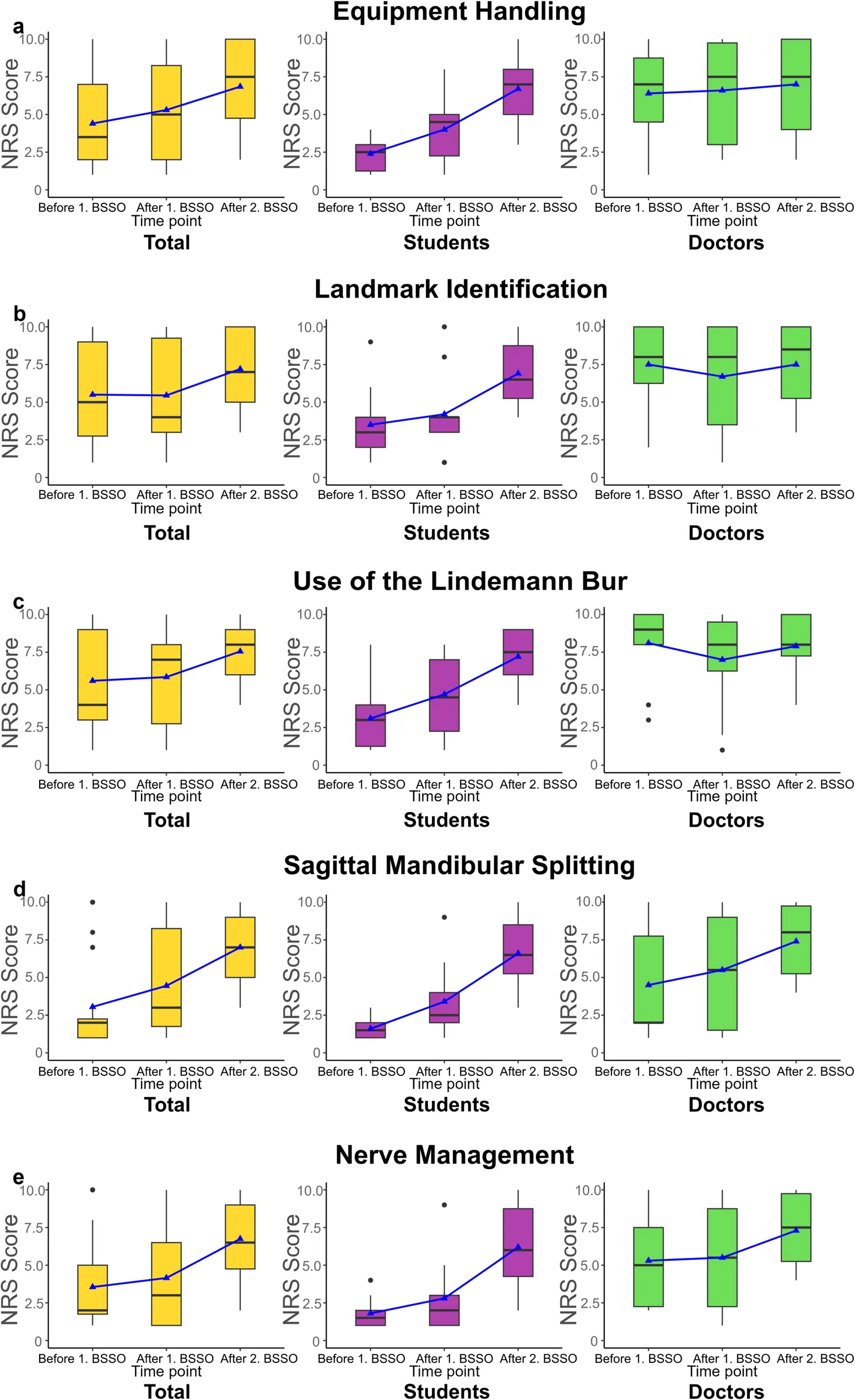
Results of the NRS in **(a)** equipment handling, **(b)** identification of landmarks, **(c)** use of a Lindemann bur, **(d)** sagittal mandibular splitting and **(e)** nerve management before the 1. BSSO, after the 1. BSSO and after the 2. BSSO. Each boxplot shows the mean value (blue triangle). The black points mark the outliers. NRS = Numeric Rating Scale

Repoted improvements in self-assessment (NRS) were observed among participants for surgical equipment handling, landmark identification, use of the Lindemann bur, sagittal mandibular splitting and nerve management (Friedman test, adj. *p* < 0.001 to 0.005). However, statistical significance was only reached after the second run, with the exception of sagittal splitting of the mandible, where a significant improvement was already observed after the first run (adj. *p* = 0.005) (Fig. 6). Subgroup analysis revealed that both students and doctors showed significant improvements across most domains. Improvements were observed in all categories for students and in all but Lindemann bur handling for doctors. Notably, after the first run, a significant improvement was observed only in students for sagittal splitting of the mandible, whereas all other domains reached significance only after the second run (Supplementary Material).

### Minimum distance between IAN and fracture surface and bad splits

**Fig. 7:**
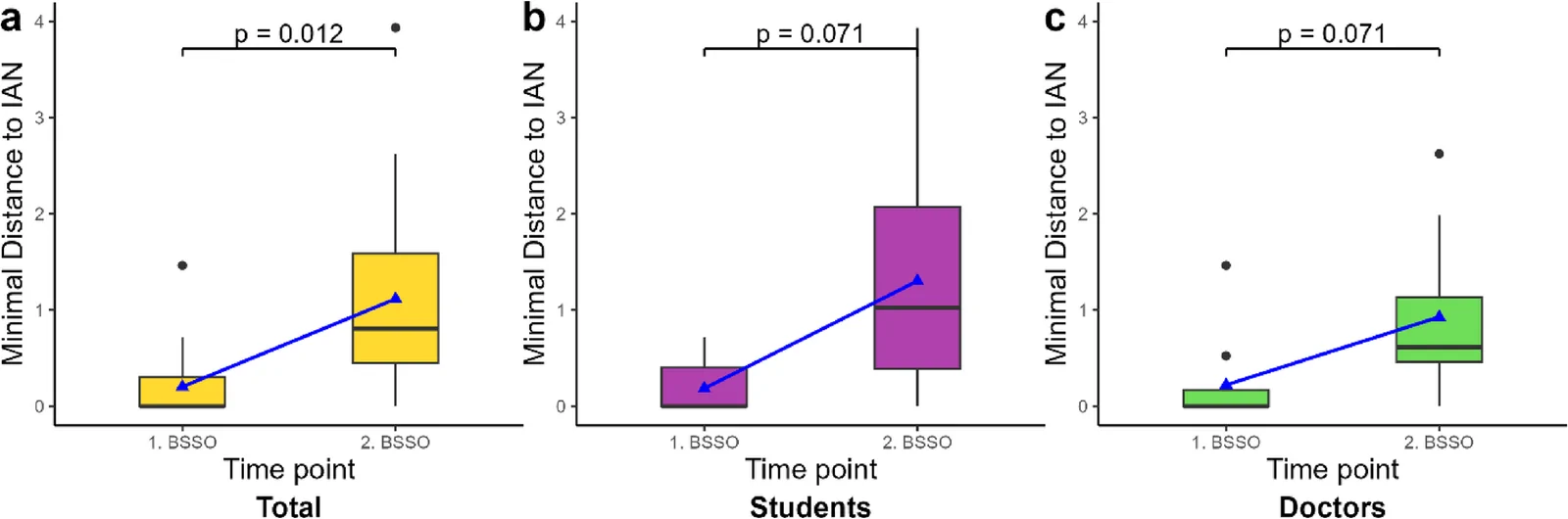
Minimal distance in mm between IAN and split surface of **a** doctors, **b** students, **c** total during the 1. BSSO and 2. BSSO. Each boxplot shows the mean value (blue triangle). The black points mark the outliers. IAN = Inferior Alveolar Nerve. The p-value (Wilcoxon signed-rank test) is shown and Holm-corrected

Calculating the minimal distance showed that, during the first trial, participants were at least 0.20 ± 0.38 mm away from the IAN, whereas during the second trial, this distance increased to 1.12 ± 1.04 mm (Wilcoxon signed-rank test, adj. *p* = 0.012; Fig. 7a). Among the students, there was an improvement from 0.18 ± 0.30 mm to 1.30 ± 1.24 mm (Wilcoxon signed-rank test, adj. *p* = 0.071; Fig. 7b). The doctors initially maintained a distance of 0.22 ± 0.47 mm, which increased to 0.93 ± 0.81 mm in the second trial (Wilcoxon signed-rank test, adj. *p* = 0.071; Fig. 7c). The interrater reliability analysis showed excellent agreement between raters, with an ICC for absolute agreement of 0.95 and an ICC for consistency of 0.94.

All models that did not fracture along the posterior or lower edge of the mandibular ramus according to Dal Pont’s surgical technique were categorised as bad splits (Figure S1). In the first run, the undesired fracture occurred 15 times; in the second run, it occurred four times (McNemar test, adj. *p* = 0.044). Among the students, this number decreased from 7 to 3 (McNemar test, adj. *p* = 0.221); among the doctors, it decreased from 8 to 1 (McNemar test, adj. *p* = 0.071).

### Likert questions and open questions

Evaluation of the Likert questions revealed generally positive perceptions of the models, particularly with regard to their clinical relevance and potential to enhance patient safety (Table 1). Both students and doctors rated the models’ practical suitability highly, although slight differences in the assessment of anatomical realism emerged across subgroups.

**Table 1:** Likert questionnaire conducted by students and doctors

Mean (± sd)	Total	Students	Doctors
I perceived the training of the BSSO on the 3D-printed mandible as realistic.	3.1 ± 0.85	2.9 ± 1.1	3.3 ± 0.48
I found the spongiosa of the 3D-printed mandible model visually realistic.	3.4 ± 0.75	3.5 ± 0.53	3.3 ± 0.95
I found the cortical bone of the 3D-printed mandible model visually realistic.	3.2 ± 0.7	3.2 ± 0.42	3.2 ± 0.92
I found the nerve of the 3D-printed mandible model visually realistic.	3.05 ± 1	3.4 ± 0.52	2.7 ± 1,25
I found the teeth of the 3D-printed mandible model visually realistic.	2.85 ± 1.04	2.6 ± 1.17	3.1 ± 0.88
I believe that BSSO training on 3D-printed mandibles increases patient safety.	3.85 ± 0.37	3.9 ± 0.32	3.8 ± 0.42
I believe that training on 3D-printed mandibles improves the outcome of BSSO.	3.8 ± 0.41	3.8 ± 0.42	3.8 ± 0.42
I found the use of the 3D-printed training model practical for clinical application.	3.65 ± 0.49	3.6 ± 0.52	3.7 ± 0.48
I believe that training on patient-specific 3D-printed mandibles is good preparation for upcoming surgeries.	3.75 ± 0.44	3.7 ± 0.48	3.8 ± 0.42
I would recommend BSSO training on 3D-printed mandibles.	3.95 ± 0.22	4 ± 0.0	3.9 ± 0.32

In the open-ended responses, participants praised the model’s realism, particularly the visual distinction between cancellous and cortical bone, which enhanced spatial anatomical understanding (Table 2). The realistic appearance, tactile feedback and the opportunity it provided for risk-free surgical practice were all positively highlighted, as were effective instrument handling and anatomical replication. Criticisms focused on the absence of a phantom mask, difficulty identifying the IAN and limited working space, which was especially challenging for less experienced users.

**Table 2:** Feedback from participants categorised as positive or negative

Positive	Negative
• theoretical instructions and personal feedback • visual and haptic distinction between cancellous and cortical bone • visual representation of the blood-filled bone • low cost • easy-to-access • patient-specific • first operative experience for beginners • realistic anatomical structures	• material entangled to bur • inadequate lighting • IAN difficult to see intraoperative • lack of tissue haemorrhage/swelling • no soft tissue • difficult view and limited working space for students

### Subgroup analysis of the doctors

A post-hoc subgroup analysis of doctors was conducted, including six participants with ≥ 5 years of clinical experience and four with < 5 years. Detailed results are presented in Supplementary Tables 1–5.

## Discussion

Three-dimensional printed anatomical models are becoming increasingly important in modern medicine as technological advancements play an expanding role in surgical training, preoperative planning and intraoperative applications [21, 23, 28, 30, 45]. Simulation-based training using patient-specific 3D models has been shown to benefit oral and maxillofacial surgery trainees and medical students by reducing operating time and blood loss and lowering the risk of repeat surgery [3, 38]. The development of surgical skills requires structured, realistic training environments. Lecture-based instruction is insufficient for developing manual skills [12]. Furthermore, digital tools such as virtual planning software and virtual reality (VR) systems can enhance the efficiency of learning and boost trainee confidence in BSSO education [4, 25, 44, 50]. However, Sohmura et al., note that VR can simulate operations only to a limited extent due to the absence of a physical environment and haptic feedback [42]. To address this, 3D-printed models have become an increasingly important component of maxillofacial training: these models provide effective hands-on experience and have been shown to improve understanding of complex anatomical structures [1, 20, 49]. 

In this context, we used a semi-professional multi-tool FDM printer to substantially reduce the material cost of producing a mandibular model, with an estimated reduction of up to 39-fold compared to previously reported approaches [6]. However, this comparison is based on material costs only and should be interpreted in light of the use of high-end industrial printing systems in the referenced study, which are associated with considerably higher overall production costs. Training using this model was associated with improved performance within a structured setting involving repeated practice and feedback. Unlike commercial products, the model can be rapidly reproduced and, in principle, adapted for patient-specific use, based on preoperative CT or CBCT data. Both patient-specific models for preoperative rehearsal and anatomically varied models for training purposes appear feasible. In the present study, a model derived from a database of healthy skulls was used to ensure consistency and controlled evaluation, while patient-specific applications represent a future direction. These benefits clearly democratise surgical teaching, especially in regions with limited resources. However, McDougall postulated that training simulators must be both reliable and valid to offer real added value in surgical training [24]. These requirements were largely met in the present study (Table 3). The jaw models could be produced reliably in large quantities and easily fixed to the phantom head, providing standardised testing conditions for all participants. In our case, a complete jaw model was produced with material costs of approximately US$4 , excluding machine-related and labor costs and requires a printing time of about 9.5 h. The 3D-printer used in the study required a one-time investment of approximately US$4300. The evaluation comprised a total of 40 BSSOs (US$160).

**Table 3:** Validity and reliability of the simulation model

	strengths	limitations
reliability	▫ standardised setting ▫ reproducible manufacturing consistency and objectiveness of measurement instrument	▫ moderate OSATS interrater-reliability
face validity	▫ realistic anatomy ▫ favourable evaluation by experienced surgeon	▫ no phantom mask ▫ no sterile cover
criterion validity	▫ -	▫ no comparison with validated simulators or actual surgical outcomes
construct validity	▫ differentiation between students and doctors ▫ doctors performed significantly better	▫ no comparison with a control cohort
content validity	▫ authentic instruments ▫ simulation of one typical complication ▫ distinctions in sawing behaviour between cortical and cancellous bone	▫ lack of soft tissue, blood vessels, haemorrhages ▫ no osteosynthesis performed ▫ limited biomechanical properties ▫ melting behaviour of PLA

In addition, the validated OSATS assessment tool enabled structured evaluation of surgical skills. Moderate agreement and good consistency were demonstrated between raters in terms of interrater reliability [18]. Overall, the OSATS-based GRS score appears to be a consistent outcome measure, while also reflecting the usual variability in rater-based assessments. Agreement might be further improved through preparatory rater training, including prior discussion and joint calibration exercises [36]. In terms of validity, our simulator demonstrated several strengths. The anatomical accuracy, for example through the use of colour-differentiated materials, a flexible nerve and the possible movement in the phantom head, contributed significantly to the face and content validity. Typical surgical complications, such as bad splits or damage to the IAN could also be simulated realistically. Real surgical instruments were also used. The distinction between cortical and cancellous bone was clearly perceptible during osteotomy.

In addition to quantifiable improvements in technical execution, the model provided an opportunity to practice surgical decision-making in more realistic anatomical conditions. For instance, the participants were tasked with selecting the most suitable osteotomy route and adapting their procedure to accommodate variability in bone structures or the positioning of nerves. The presence of the fracture line and the potential to identify erroneous osteotomy lines further enabled participants to experience the immediate effects of technical inaccuracies. Clearly, this learning effect cannot be achieved through theoretical instruction alone [37].

The evaluation of the NRS provides revealing information about participants’ subjective assessment of their gain in competence (Fig. 6). Initially, doctors tended to overestimate their skills, demonstrating high confidence at baseline [19]. However, an increase in self-assessment was evident over the course of the study, presumably due to the repetition effect and improved adaptation to the simulation environment. In contrast, the students reported continuous improvement in their skills across both sessions. It can be concluded that this group benefited from the training provided by the model. The findings suggest that novice participants experience a steeper learning curve than those with prior experience. This highlights the effectiveness of the training environment, particularly in training novices.

Although a reduction in the number of bad splits was observed, the underlying causes were not systematically analysed and thus can only be discussed based on procedural observations. Potential contributing factors included insufficient osteotomy depth, deviation from the intended osteotomy trajectory and suboptimal chisel positioning or angulation, as well as premature or excessive force application during splitting.

As only the osteotomy and mandibular splitting were tested in this study, it is not possible to draw any conclusions about the osteosynthesis behaviour of the model. Shujaat et al., demonstrated that models fabricated using FDM printing are competitive with alternative methods when it comes to drilling screw holes. However, they were found to be significantly inferior in terms of user experience when it came to inserting and removing screws [41]. It must be acknowledged that the mechanical properties are partly determined by the orientation of the model on the print bed, with this effect being more pronounced in FDM printing than in PolyJet technology due to the thicker layers used in FDM [40]. In contrast to native mandibular bone, which exhibits anisotropic fracture behaviour guided by cortical boundaries and trabecular architecture, the FDM-printed PLA model shows a more layer-dependent fracture pattern [16, 48]. As described in the methods section, the print orientation was intentionally selected to align the mandibular ramus with the printing layers in order to facilitate a controlled and reproducible splitting plane. While this design enables consistent training conditions, it results in a more predefined fracture pathway and may limit the physiological realism of tactile feedback during osteotomy and sagittal splitting. Therefore, it can be deduced that developing a universally applicable mandibular model using the FDM printing process is challenging; rather, customisation for specific operations is required.

Other technical challenges must also be considered, such as PLA melting under excessive pressure when using rotating instruments and an increased risk of slipping due to the smooth PLA surface. In addition, the mechanical behaviour of 3D-printed models is not determined by the material alone, but also by printing parameters and structural design, such as cortical thickness, internal infill and infill geometry [15]. Therefore, the effective stiffness may differ from that of mandibular cortical bone, which can influence the perceived resistance and haptic feedback during osteotomy and splitting. Alternative engineering filaments such as PC, ABS, PETG or Nylon may improve biomechanical realism [26]. However, their use must be balanced against practical limitations, such as cost, printability and material-specific constraints. Future studies should investigate alternative materials and optimised printing parameters to improve the realism of 3D-printed surgical training models.

Other limitations should also be considered. Qualitative participant feedback highlighted several important limitations regarding realism, including the absence of soft tissue, lack of bleeding simulation, difficulty in identifying the IAN intraoperatively and restricted working space. These aspects are relevant, as they represent critical components of real BSSO procedures that influence both surgical orientation and decision-making. Their absence may reduce the fidelity of the simulation and limit the extent to which procedural skills can be fully translated to the clinical setting. Additional elements such as a sterile cover and a phantom mask could improve the level of realism. The latter would be technically feasible, but it cannot be integrated into the current test setup due to video recording issues. The present study provides evidence for face and construct validity, as reflected by perceived realism and the superior performance of more experienced participants. However, no conclusions can be drawn regarding educational transfer to clinical practice or patient outcomes. Criterion validity, including comparison with established simulators or real surgical performance, was not assessed and remains to be investigated in future studies.

While technical aspects such as printing orientation and material properties influence the biomechanical realism of mandibular models, the ultimate goal is to enhance surgical technique and improve patient outcomes. In this context, handling delicate structures such as the IAN is crucial. As the IAN was embedded directly within the cancellous structure, without a surrounding cortical layer, some degree of tactile feedback present in real surgery is reduced, particularly the clear differentiation between the cortical boundary and the surrounding bone. This may lead to an underestimation of the perceived proximity to the nerve during training, as no distinct anatomical landmark is present. Since working with a chisel can lead to compression of the cancellous bone or neurosensory disturbances caused by traction on the IAN or its accompanying vessels, it is important to maintain sufficient distance [27]. However, the current literature provides no specific information on the average distance between fracture surfaces and the IAN, as this varies depending on the anatomy of the mandibular canal and cortical bone. This should be considered in light of the fact that the anatomical configuration in this study was derived from a CT dataset of a real patient and therefore represents a single anatomical scenario without capturing the full range of inter-individual variability of the IAN. Accordingly, the observed performance reflects a controlled training scenario rather than clinical complexity.

The mandibular canal is typically surrounded by a dense cortical bone layer that provides important tactile feedback and surgical orientation. Due to limitations of the slicing workflow implemented in PrusaSlicer, this cortical canal layer could not be reproduced and the nerve was instead embedded within cancellous bone. Consequently, the model offers reduced tactile differentiation, which may affect the perception of nerve proximity during training. This limitation should be considered when interpreting the realism and transferability of the model.

Nevertheless, participants demonstrated a significant tendency towards nerve-sparing surgery and increased awareness of critical anatomical structures in the second round (Fig. 7). Despite the nominal scanner resolution being high, the effective measurement uncertainty is primarily influenced by registration accuracy and operator-dependent segmentation of the fracture surface. However, in this study, the use of two independent raters demonstrated excellent interrater reliability, indicating a high degree of consistency in the segmentation process and suggesting that operator-dependent variability was limited.

A major limitation of this study is the absence of a control group, which prevents isolation of the specific effects of the 3D-printed model. The intervention was deliberately designed as a bundled educational approach, including model use, instructional video and expert feedback, which reflects a realistic training scenario, but precludes attribution of observed improvements to any single component. In particular, the introduction of video instruction and expert feedback after the first training run likely contributed to an expected learning effect, thereby influencing performance in the second run. A controlled study design, for example including comparison with a commercially available model, would be required to disentangle these effects.

Future studies should not only investigate the transfer of training outcomes to real surgical performance and the development of patient-specific model fabrication, but also the realistic integration of soft tissue and bleeding. Despite the increased technical effort and costs, the incorporation of silicone or gel-based soft tissue layers via manual post-processing or multi-material printing has the potential to simulate mucosal and periosteal resistance and enhance instrument-tissue interaction fidelity. Incorporating pathological features, such as fluid-filled cysts lined with TPU printing material or variable bone densities, could improve the realism of the simulation. The ultimate objective of 3D-printing in surgical training is to enhance long-term patient outcomes through the augmentation of surgical precision and informed decision-making, not simply to replicate anatomical structures. The present model demonstrates that strategically designing and systematically implementing cost-effective simulation tools can offer high added value in terms of education, especially if they are tailored to beginners’ learning curves and specific surgical challenges.

## Conclusion

This model, embedded in a structured training environment, could serve as a realistic and cost-effective training tool to support the development of surgical skills in BSSO. It may also have the potential to support future, patient-specific rehearsal of upcoming operations in oral and maxillofacial surgery. The underlying approach could potentially be extended to other anatomical structures and surgical specialties. However, further studies are required to validate the simulator and to assess its impact on real-life surgical performance.

## Supplementary Information

Supplementary Material 1.

Supplementary Material 2.

Supplementary Material 3.

Supplementary Material 4.

## Data Availability

The data presented in this study are available on request from the corresponding author.
