# Supplementary material for: Evaluation of structured training in osteotomy and splitting for BSSO using a 3D-printed mandibular model
Source: BMC Med Educ. 2026 Jul 9;26:1359. doi: 10.1186/s12909-026-09748-w (PMC13499296; doi:10.1186/s12909-026-09748-w)
Supplement: Supplementary file 1 [file 12909_2026_9748_MOESM4_ESM.docx]

# Tables

**Table S1 -** comparison of GRS scores stratified by experience group (<5 years vs ≥5 years)

| subgroup | 1. BBSO (Mean ± SD) | 2. BBSO (Mean ± SD) | Δ (2. BSSO – 1. BSSO) | p-value |
| --- | --- | --- | --- | --- |
| < 5 years | 26.33 ± 3.47 | 30.08 ± 1.94 | +3.75 | 0.181 |
| ≥5 years | 26.75 ± 3.42 | 29.38 ± 3.50 | +2.63 | 0.031 |

**Table S2 -** comparison of NRS scores stratified by experience group (<5 years vs ≥5 years)

| category | subgroup | T1 mean ± sd | T2 mean ± sd | T3 mean ± sd | p-value | post-hoc comparisons p-value |
| --- | --- | --- | --- | --- | --- | --- |
| Equipment | <5 years | 7.25 ± 1.26 | 6.75 ± 3.59 | 7.00 ± 2.94 | 0.526 | T1 vs T2: 1.000; T2 vs T3: 0.773 |
| Equipment | ≥5 years | 5.83 ± 4.02 | 6.50 ± 3.78 | 7.00 ± 3.69 | 0.050 | T1 vs T2: 0.174; T2 vs T3: 0.149 |
| Landmarks | <5 years | 8.00 ± 1.83 | 6.25 ± 4.11 | 7.25 ± 3.40 | 0.150 | T1 vs T2: 0.371; T2 vs T3: 0.174 |
| Landmarks | ≥5 years | 7.17 ± 3.49 | 7.00 ± 3.69 | 7.67 ± 2.73 | 0.156 | T1 vs T2: 1.000; T2 vs T3: 0.346 |
| Lindemann bur | <5 years | 8.50 ± 0.58 | 6.00 ± 3.37 | 7.25 ± 2.50 | 0.148 | T1 vs T2: 0.174; T2 vs T3: 0.371 |
| Lindemann bur | ≥5 years | 7.83 ± 3.37 | 7.67 ± 3.20 | 8.33 ± 2.34 | 0.441 | T1 vs T2: 1.000; T2 vs T3: 0.346 |
| Splitting | <5 years | 1.75 ± 0.50 | 3.75 ± 2.99 | 6.25 ± 2.22 | 0.039 | T1 vs T2: 0.269; T2 vs T3: 0.125 |
| Splitting | ≥5 years | 6.33 ± 3.93 | 6.67 ± 4.41 | 8.17 ± 2.56 | 0.062 | T1 vs T2: 0.586; T2 vs T3: 0.181 |
| Nerve handling | <5 years | 3.75 ± 2.06 | 3.50 ± 2.38 | 6.00 ± 1.83 | 0.307 | T1 vs T2: 1.000; T2 vs T3: 0.181 |
| Nerve handling | ≥5 years | 6.33 ± 3.50 | 6.83 ± 3.87 | 8.17 ± 2.56 | 0.062 | T1 vs T2: 0.586; T2 vs T3: 0.174 |

**Table S3 -** comparison of the Likert questionnaire stratified by experience group (<5 years vs ≥5 years)

| category | <5 years (m ± sd) | ≥5 years (m ± sd) |
| --- | --- | --- |
| I perceived the training of the BSSO on the 3D-printed mandible as realistic. | 3.25 ± 0.50 | 3.33 ± 0.52 |
| I found the spongiosa of the 3D-printed mandible model visually realistic. | 3.25 ± 1.50 | 3.33 ± 0.52 |
| I found the cortical bone of the 3D-printed mandible model visually realistic. | 3.75 ± 0.50 | 2.83 ± 0.98 |
| I found the nerve of the 3D-printed mandible model visually realistic. | 3.00 ± 1.41 | 2.50 ± 1.22 |
| I found the teeth of the 3D-printed mandible model visually realistic. | 2.75 ± 1.26 | 3.33 ± 0.52 |
| I believe that BSSO training on 3D-printed mandibles increases patient safety. | 4.00 ± 0.00 | 3.67 ± 0.52 |
| I believe that training on 3D-printed mandibles improves the outcome of BSSO. | 3.75 ± 0.50 | 3.83 ± 0.41 |
| I found the use of the 3D-printed training model practical for clinical application. | 3.75 ± 0.50 | 3.67 ± 0.52 |
| I believe that training on patient-specific 3D-printed mandibles is good preparation for upcoming surgeries. | 4.00 ± 0.00 | 3.67 ± 0.52 |
| I would recommend BSSO training on 3D-printed mandibles. | 4.00 ± 0.00 | 3.83 ± 0.41 |

**Table S4 -** comparison of IAN distance measurements stratified by experience group (<5 years vs ≥5 years)

| subgroup | 1. BBSO (Mean ± SD) | 2. BBSO (Mean ± SD) | Δ (2. BSSO – 1. BSSO) | p-value |
| --- | --- | --- | --- | --- |
| < 5 years | 0.13 ± 0.26 | 1.05 ± 1.06 | +0.92 | 0.250 |
| ≥5 years | 0.28 ± 0.59 | 0.85 ± 0.69 | +0.57 | 0.156 |

**Table S5 -** comparison of bad splits stratified by experience group (<5 years vs ≥5 years)

| subgroup | 1. BBSO (n/N) | 2. BBSO (n/N) | p-value |
| --- | --- | --- | --- |
| <5 years | 3/4 | 0/4 | 0.248 |
| ≥5 years | 5/6 | 1/6 | 0.134 |

# Supplementary Figures

## Figure S1

**Figure S1.** Representative visualisation of desired and undesired fracture patterns based on mandibular segments reconstructed from scans in Blender. (**a)** ideal split pattern fracture along the posterior or lower edge of the mandibular ramus according to Dal Pont's surgical technique (**b**) a bad split characterised by incomplete or irregular splitting. The dashed line represents the intended fracture and osteotomy line.
